# Underwater Pressure Lumen Expansion: A Novel Method to Overcome Lumen Collapse in Submucosal Endoscopy and Animal Endoscopic Full‐thickness Resection Models

**DOI:** 10.1002/deo2.70195

**Published:** 2025-09-10

**Authors:** Hironari Shiwaku, Akio Shiwaku, Seiya Sato, Kosuke Yamauchi, Suguru Hasegawa

**Affiliations:** ^1^ Department of Gastroenterological Surgery Fukuoka University Faculty of Medicine Fukuoka Japan

**Keywords:** endoscopic full‐thickness resection (EFTR), hydraulic pressure, luminal collapse, underwater endoscopy, water pressure technique

## Abstract

In endoscopic full‐thickness resection (EFTR), luminal collapse can occur due to communication between the gastrointestinal lumen and peritoneal cavity, making visualization and procedural continuity difficult. We propose the underwater pressure lumen expansion (UPLE) method, in which hydraulic pressure is applied in a fluid‐filled environment to re‐expand the collapsed lumen, thereby restoring visualization and allowing continued endoscopic manipulation. The UPLE method was applied during peroral endoscopic tumor resection in a clinical case and in an animal model of EFTR, and its utility was successfully demonstrated.

## 1 Introduction

**FIGURE 1:**
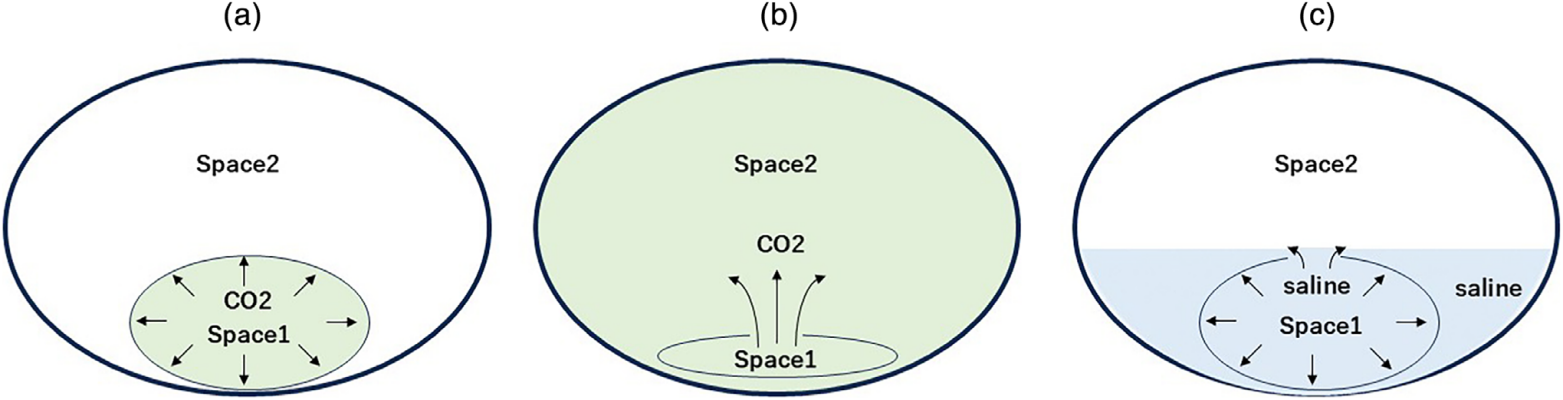
Overview of the underwater pressure lumen expansion (UPLE) method. (a) In therapeutic procedures using flexible endoscopy, visualization and maneuverability are achieved by insufflating the target space (Space 1) with CO_2_. (b) When Space 1 communicates with an adjacent space (Space 2) and collapses during the procedure, insufflation alone becomes insufficient, leading to loss of expansion and difficulty continuing the procedure. (c) By immersing both Space 1 and Space 2 in an underwater environment and applying hydraulic pressure to Space 1, re‐expansion of the lumen is achieved, allowing for continued visualization and endoscopic manipulation. This technique is termed the UPLE (underwater pressure lumen expansion) method.

During endoscopic full‐thickness resection (EFTR), full‐thickness incision of the gastric wall establishes communication between the gastric lumen (Space 1) and the peritoneal cavity (Space 2), leading to collapse of the intraluminal space (Figure 1a,b). This collapse significantly hinders visualization and maneuverability, and has long been recognized as the most critical technical obstacle in EFTR. Until now, no effective solution has been established to address this problem.

However, we found that by immersing both Space 1 and Space 2 in a fluid‐filled environment and applying hydraulic pressure within Space 1, re‐expansion of the lumen could be achieved, thereby restoring visualization and allowing continued endoscopic manipulation (Figure 1c). We have named this technique the Underwater Pressure Lumen Expansion (UPLE) method.

In this report, we describe a case in which the UPLE method was applied during peroral endoscopic tumor resection (POET) ^[1]^ in a human subject. We also demonstrate the reproducibility of this technique in an animal model of gastric EFTR.

### 1.1 Case Reports

The UPLE method was applied in one patient undergoing POET, with written informed consent obtained from the patient. The treatment was conducted in accordance with the principles of the Declaration of Helsinki.

The UPLE method was also evaluated in a porcine model of gastric endoscopic full‐thickness resection. All animal procedures were approved by the Animal Care and Use Committee of the Kobe Medical Device Development Center (Approval No.: IVT25‐39) and conducted in accordance with institutional guidelines and the Animal Research: Reporting of In Vivo Experiments guidelines.

### 1.2 Case 1

**FIGURE 2:**
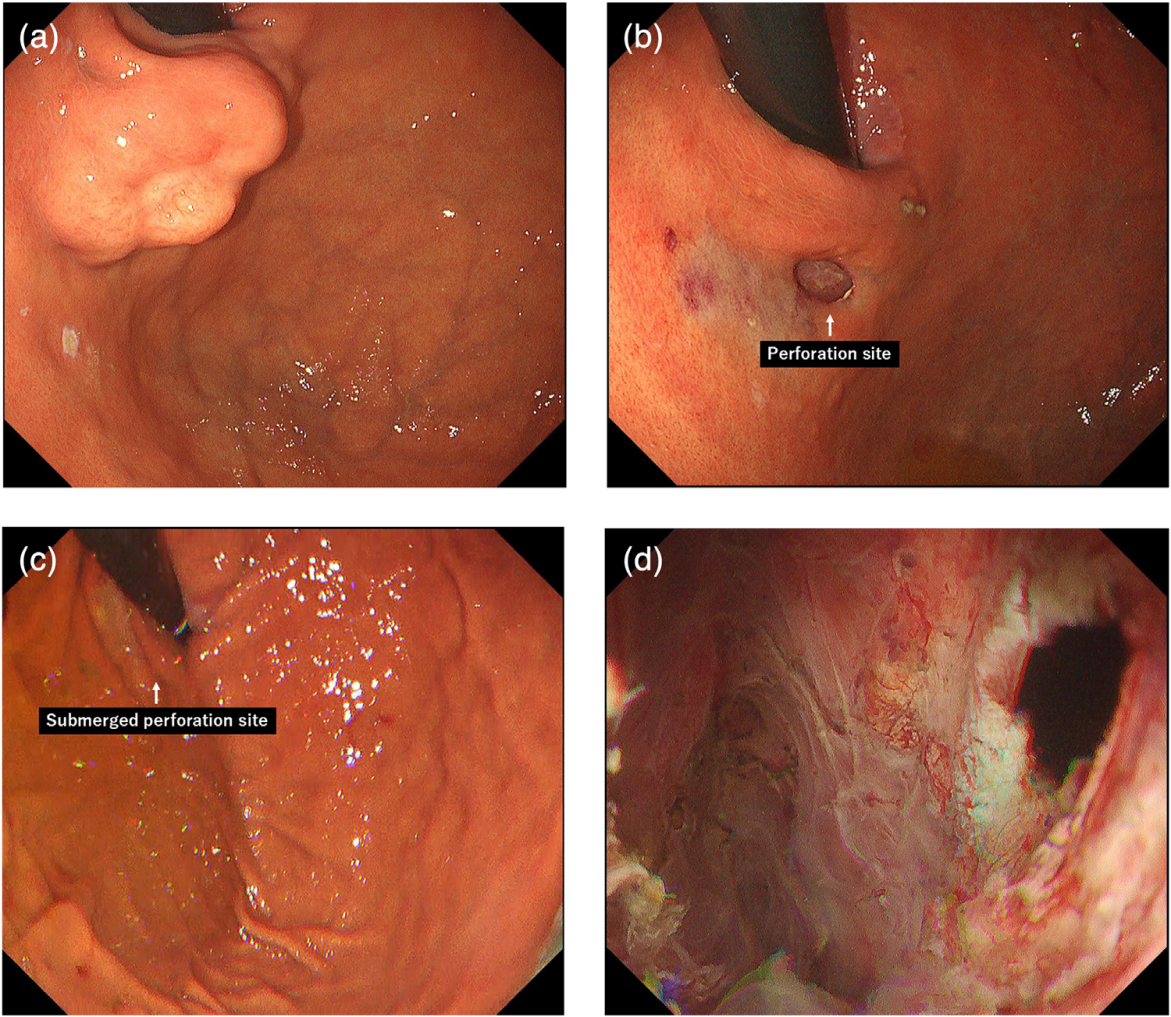
Restoration of the endoscopic view using the underwater pressure lumen expansion (UPLE) method for submucosal tunnel collapse due to mucosal perforation during peroral endoscopic tumor resection (POET) in a human case. (a) A 3‐cm submucosal tumor was identified on the posterior wall of the upper gastric body near the esophagogastric junction, with scarring on its apex due to prior boring biopsy. (b) During POET, partial mucosal injury occurred at the scar site, resulting in mucosal perforation. (c) Normal saline was infused into the submucosal tunnel (Space 1), submerging both the gastric lumen (Space 2) and the perforation site at the esophagogastric junction. (d) Endoscopic view of the submucosal tunnel after tumor resection. CO_2_ insufflation failed to adequately expand the tunnel, but satisfactory expansion was achieved using the UPLE method.

A woman in her 70s was found to have a submucosal tumor about 3 cm in diameter at the esophagogastric junction. A prior boring biopsy at another hospital diagnosed it as a leiomyoma (Figure 2a). She was referred to our institution, and POET was performed in the supine position. A mucosal incision was made in the lower thoracic esophagus, and the endoscope was inserted into the submucosal layer. Scar tissue from the prior biopsy was noted between the mucosa and the tumor, where mucosal perforation occurred in the posterior wall of the upper gastric body, resulting in communication between the submucosal tunnel (Space 1) and the gastric lumen (Space 2) (Figure 2b).

After removal of the tumor, hemostasis was attempted. However, expansion of the submucosal tunnel (Space 1) could not be maintained, and adequate visualization became difficult.

At this point, the UPLE method was introduced(Supporting Information 1). Using the waterjet function of the endoscope with the JW‐3 waterjet system (FujiFilm Japan) at an infusion rate of about 200 mL/min, normal saline was infused into the submucosal tunnel (Space 1), and both the gastric lumen (Space 2) and the mucosal perforation site at the esophagogastric junction were submerged (Figure 2c). When hydraulic pressure was applied within the submucosal tunnel (Space 1), the space re‐expanded, allowing for visualization, within less than 1 min after initiation of the UPLE method (Figure 2d). After confirming complete tumor resection and the absence of exposed vessels, the mucosal injury and incision sites were closed with endoclips, and the procedure was completed. The patient was discharged without complications.

### 1.3 Case 2

To demonstrate the ERBE HYBRIDknifeflex and Argon Plasma Coagulation (APC) probe, we performed gastric endoscopic submucosal dissection (ESD), per‐oral endoscopic myotomy, and APC coagulation using a porcine gastrointestinal model. Subsequently, we investigated the feasibility of applying the UPLE method to gastric EFTR (Supporting Information 2).

**FIGURE 3:**
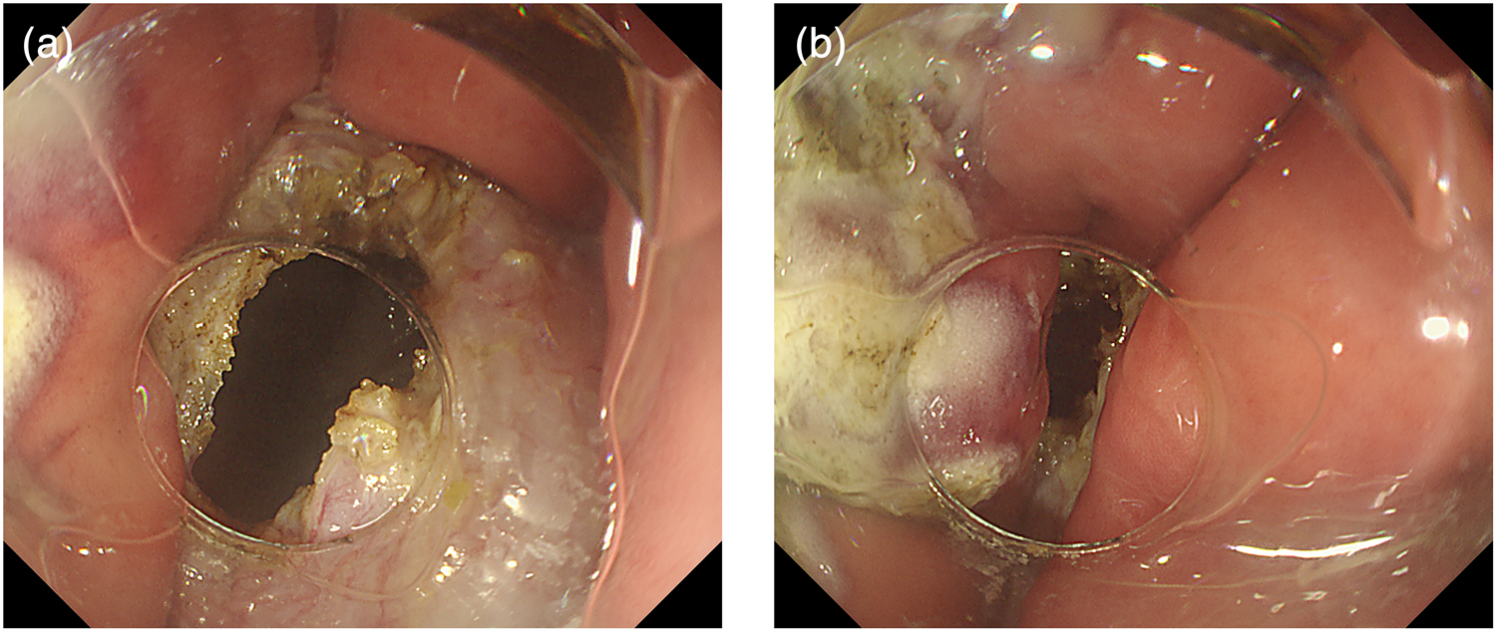
Restoration of visualization and endoscopic operability using the underwater pressure lumen expansion (UPLE) method for gastric lumen collapse during endoscopic full‐thickness resection (EFTR) in an animal model. (a, b) Full‐thickness resection was performed on the greater curvature of the gastric body. Shortly afterward, CO_2_ insufflation failed to sufficiently distend the gastric lumen, resulting in instability of endoscopic manipulation.

**FIGURE 4:**
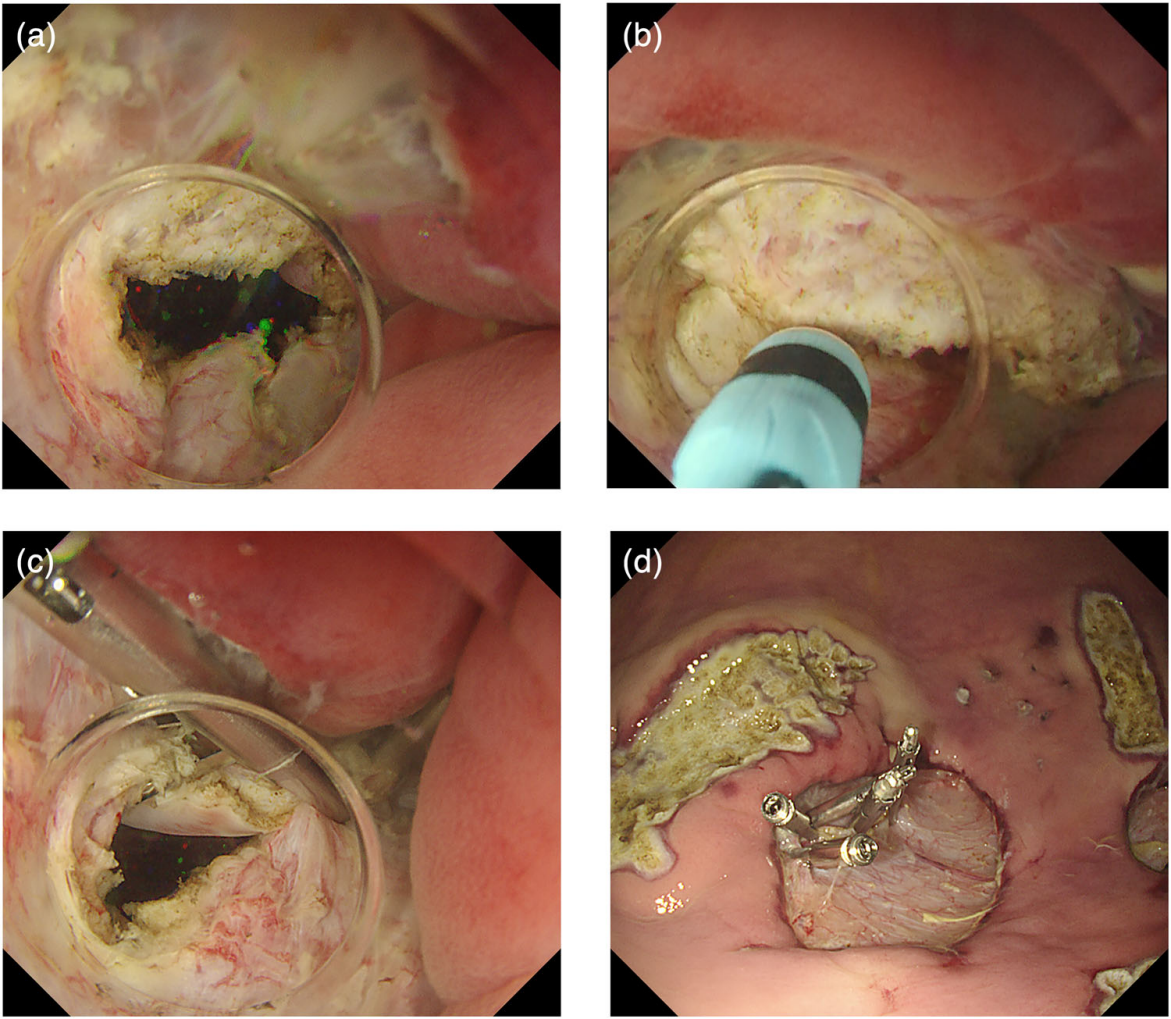
Restoration of visualization and endoscopic operability using the underwater pressure lumen expansion (UPLE) method for gastric lumen collapse during endoscopic full‐thickness resection (EFTR) in an animal model. (a–c) After restoring the visual field using the UPLE method, endoscopic operability was regained, allowing for additional full‐thickness resection followed by wound closure with endoscopic clips. (d) Following complete closure of the wound, CO_2_ insufflation successfully redistended the gastric lumen, restoring visibility and operability to pre‐resection levels.

After performing ESD on the greater curvature of the gastric body, we incised the muscularis propria at the ulcer base, thereby creating communication between the gastric lumen (Space 1) and the peritoneal cavity (Space 2) (Figure 3a). As a result, the stomach collapsed (Figure 3b), and continuation of the procedure became difficult. Using the EIP2 (ERBE, Germany) at an infusion rate of 150 mL/min, approximately 500 mL of normal saline was infused into the gastric lumen and peritoneal cavity. Applying hydraulic pressure within the gastric lumen caused the gastric wall to re‐expand and the visual field to be restored (Figure 4a), enabling further extension of the muscular incision (Figure 4b) and closure of the incision site with endoscopic clips (Figure 4c). After completion of closure, all intragastric saline was aspirated, and re‐expansion of the stomach with CO_2_ insufflation was confirmed (Figure 4d). Ultimately, a total of approximately 1 liter of normal saline was used.

## 2 Discussion

In recent years, reports have increasingly highlighted the utility of endoscopic procedures performed under underwater conditions. Among them, Yahagi et al.,^[^ ^2^, ^3^ ^]^ demonstrated that the water pressure method in duodenal ESD improves visualization and facilitates access to the submucosal layer.

In contrast, no previous reports have applied underwater pressure techniques to address luminal collapse during EFTR. In this study, we applied the UPLE method to two scenarios: submucosal tunnel collapse during POET in a human case and gastric lumen collapse following EFTR in a porcine model. In both settings, in the first POET case, although we had anticipated the possibility of mucosal perforation and had considered this as one of the potential measures in the event of lumen collapse within the submucosal tunnel, the UPLE method was not planned from the outset.

In the second case, as it was an animal experiment conducted to evaluate the usefulness of the UPLE method, it had been prepared in advance. In both cases, the UPLE method was effective in restoring the endoscopic view and ensuring procedural continuity.

The main cause of space collapse (Space 1) during endoscopic procedures is the inability to maintain intraluminal pressure due to gas (CO_2_) leakage, which leads to the collapse of the surrounding walls. CO_2_, in particular, has low density and viscosity and easily escapes through even small openings. Thus, when Space 1 communicates with Space 2, insufflation cannot sustain pressure, resulting in inadequate expansion (Figure 1b).

In contrast, normal saline is a dense, incompressible fluid that, when infused into a cavity, uniformly expands its walls via hydrostatic pressure. Unlike gas, fluids do not rapidly escape through small defects, allowing stable pressure maintenance. Moreover, the UPLE method involves not only filling Space 1 but also Space 2 with fluid, minimizing the pressure gradient between compartments and preventing collapse (Figure 1c). Compared to gaseous insufflation, water pressure provides a more homogeneous and sustained expansion force, which contributes significantly to securing the visual field and enhancing procedural safety.

During the UPLE method, continuous water infusion is performed until the visual field is restored, after which water is infused as needed. The sufficiency of the infused fluid can be determined endoscopically by three points: [1] confirming that Space 1 is under water immersion, [2] checking whether Space 1 expands and the visual field is restored by water pressure, and [3] if the situation allows, inspecting the area around the site of communication between Space 1 and Space 2 to confirm that Space 2 is also under water immersion.

Despite its potential, several challenges need to be addressed before UPLE can be widely applied in clinical practice. Residual fluid may remain in the peritoneal cavity after EFTR, but previous studies suggest that saline or Ringer's solution is absorbed within a few days ^[^ ^4^, ^5^, ^6^, ^7^, ^8^ ^]^. However, to avoid the risk of abdominal compartment syndrome that may result from excessive fluid infusion, close monitoring and drainage when necessary are essential. Regarding infection, the risk is anticipated to be minimal in upper gastrointestinal procedures if closure is secure, whereas procedures involving the lower gastrointestinal tract may carry a higher risk and require careful case selection ^[^ ^9^, ^10^ ^]^. Additional supporting discussion on these points is provided in Supporting Information (Supporting Information 3).

As a limitation, the method relies on underwater visualization; therefore, in cases of significant bleeding, visibility may be compromised, potentially limiting operability.

The UPLE method holds promise as a simple, endoscopy‐only rescue technique for overcoming luminal collapse during EFTR. Larger prospective studies are needed to further assess the safety, efficacy, indications, and limitations of the UPLE method in clinical practice.

## Conflicts of Interest

We report receiving support from Amco Inc. for animal experiments related to this study. The authors declare no other conflict of interest.

## Supporting information




**Supporting File 1**: deo270195‐sup‐0002‐SuppMat.docx.

**Video S1** A video showing the novel UPLE method described in this report. Supporting Information 1 is a video demonstrating the application of the UPLE method in POET (human model). Supporting Information 2 is a video demonstrating the application of the UPLE method in gastric EFTR (porcine model). Supporting Information 3 provides additional discussion on residual fluid absorption and infection risk that may be anticipated in clinical applications.
