# Supplementary material for: Underwater Pressure Lumen Expansion: A Novel Method to Overcome Lumen Collapse in Submucosal Endoscopy and Animal Endoscopic Full‐thickness Resection Models
Source: DEN Open. 2025 Sep 10;6(1):e70195. doi: 10.1002/deo2.70195 (PMC12422953; doi:10.1002/deo2.70195)
Supplement: Supplementary file 1 — Supporting File 1: deo270195‐sup‐0002‐SuppMat.docx. [file DEO2-6-e70195-s001.docx]

**Supporting Information 3.**

**Detailed Discussion on Residual Fluid Absorption and Infection Risk**

**■Residual Fluid Absorption**

Despite its potential, several challenges must be addressed before UPLE can be widely applied in clinical practice.

First, a significant amount of saline may remain in the peritoneal cavity after closure of the gastric lumen. In procedures such as Laparoscopic and Endoscopic Cooperative Surgery, such residual fluid can be aspirated via the laparoscopic route. However, when EFTR is performed endoscopically alone, 1–2 liters of saline may remain intraperitoneally.

Regarding this concern, previous study offer some insight. Shear et al. (1) reported that saline introduced into the peritoneal cavity is absorbed at a rate of approximately 30–60 mL/hour, suggesting that several hundred milliliters of residual fluid may be spontaneously absorbed within 1–2 days. Hart et al. (2)reported that in gynecologic laparoscopic surgery cases, 1 liter of normal saline was infused into the peritoneal cavity at the end of the procedure, and the amount of fluid recovered over time was measured via drainage. As a result, they found that approximately 16 hours after surgery, the entire volume had been absorbed and no fluid could be retrieved from the drain.

However, saline is not entirely harmless—some reports suggest that it may induce peritoneal adhesions. Moreover, certain studies have indicated that Ringer’s solution may be safer than normal saline in terms of the risk of promoting adhesions (3) (4). Muzii et al. (5) demonstrated that 300 mL of lactate Ringer’s solution introduced during laparoscopic surgery was substantially absorbed within 96 hours postoperatively, indicating that crystalloids are likely to dissipate over time.

Whether to use normal saline or alternative solutions such as Ringer’s solution in the UPLE method remains inconclusive at this time. Nevertheless, these findings suggest that transient retention of saline or Ringer’s solution in the peritoneal cavity may be absorbed, provided the volume does not reach a level that risks inducing abdominal compartment syndrome. Accordingly, during the UPLE method, close communication with the anesthesiologist and periodic abdominal palpation, as performed during peroral endoscopic myotomy, are necessary to ensure that abdominal compartment syndrome has not developed. In cases where intra-abdominal pressure becomes significantly elevated, therapeutic drainage—such as suction of the peritoneal cavity (Space 2) before complete closure of the lumen (Space 1), or percutaneous paracentesis—should be considered. However, further validation is warranted to assess the effects of long-term fluid retention in humans.

**■Infection Risk**

Another important consideration is the potential risk of infection. Narula et al. (6) reported that even when gastric bacteria entered the peritoneal cavity during transgastric access in bariatric surgery, no infectious complications occurred if proper closure was ensured. Conversely, Navez et al. (7) reported cases of peritonitis and abscess formation due to closure failure in transcolonic procedures using a porcine model, indicating a higher risk of infection in lower gastrointestinal approaches.

Taken together, these findings suggest that in upper gastrointestinal applications, the risk of infection from residual saline is minimal if secure closure is achieved.

However, even in upper gastrointestinal cases, such as gastric EFTR, where the peritoneal cavity (Space 2) is a sterile space and the application of the UPLE method is expected to cause the contents of Space 1 to flow into Space 2, the gastrointestinal lumen (Space 1) should be thoroughly irrigated in advance.

In contrast, procedures involving the lower gastrointestinal tract may carry a higher risk of postoperative peritonitis, requiring careful case selection.

**■Summary**

Based on these considerations, the currently anticipated indications for the UPLE method are cases in endoscopic procedures such as submucosal endoscopy or full-thickness resection where luminal collapse significantly interferes with visualization and continuation of the procedure, particularly in the esophagus, stomach, and duodenum. It is considered useful in cases with a large communication between the lumen and the extraluminal space, where the risk of collapse is high. Contraindications are generally in line with those for submucosal endoscopy or EFTR; however, as the risk of inducing peritonitis is presumed to be higher in the lower gastrointestinal tract, indications in this setting should be considered with caution.

**Reference：**

1. Shear L, Swartz C, Shinaberger JA, Barry KG. KINETICS OF PERITONEAL FLUID ABSORPTION IN ADULT MAN. N Engl J Med. 1965;272:123-7.

2. R. Hart, Magos A. Laparoscopically instilled fluid: the rate of absorption and the effects on patient discomfort and fluid balance. Gynaecological Endoscopy. 1996;5(5):287-91.

3. Cwalinski J, Staniszewski R, Baum E, Jasinski T, Mackowiak B, Breborowicz A. Normal saline may promote formation of peritoneal adhesions. Int J Clin Exp Med. 2015;8(6):8828-34.

4. Elkelani OA, Molinas CR, Mynbaev O, Koninckx PR. Prevention of adhesions with crystalloids during laparoscopic surgery in mice. J Am Assoc Gynecol Laparosc. 2002;9(4):447-52.

5. Muzii L, Bellati F, Manci N, Zullo MA, Angioli R, Panici PB. Ringer's lactate solution remains in the peritoneal cavity after laparoscopy longer than expected. Fertil Steril. 2005;84(1):148-53.

6. Narula VK, Hazey JW, Renton DB, Reavis KM, Paul CM, Hinshaw KE, et al. Transgastric instrumentation and bacterial contamination of the peritoneal cavity. Surg Endosc. 2008;22(3):605-11.

7. Navez J, Yeung R, Remue C, Descamps C, Navez B, Gigot JF, et al. Acute-phase response in pigs undergoing laparoscopic, transgastric or transcolonic notes peritoneoscopy with us or eus exploration. Acta Gastroenterol Belg. 2012;75(1):28-34.
