# Supplementary material for: Underwater Pressure Lumen Expansion: A Novel Method to Overcome Lumen Collapse in Submucosal Endoscopy and Animal Endoscopic Full‐thickness Resection Models
Source: DEN Open. 2025 Sep 10;6(1):e70195. doi: 10.1002/deo2.70195 (PMC12422953; doi:10.1002/deo2.70195)
Supplement: Supplementary file 2 — Video S1 A video showing the novel UPLE method described in this report. Supporting Information 1 is a video demonstrating the application of the UPLE method in POET (human model). Supporting Information 2 is a video demonstrating the application of the UPLE method in gastric EFTR (porcine model). Supporting Information 3 provides additional discussion on residual fluid absorption and infection risk that may be anticipated in clinical applications. [file DEO2-6-e70195-s002.docx]

Supporting videos can be downloaded here:

[Supporting information 1.mp4](https://wiley-my.sharepoint.com/:v:/p/yikegami/EaaJAz3Qbc9Hs4LkIggrP-gBEB6KADmYI8KN4_2Db-twtQ?e=d1eZ2Y)

[Revised Supporting information 2.mp4](https://wiley-my.sharepoint.com/:v:/p/yikegami/ERf36nUEEDhEhyrj81h_Vc0BFfQiskTQ_hc3wwk-FWh5ew?e=FA2eem)
